# Genome-wide BAC-end sequencing of *Cucumis melo *using two BAC libraries

**DOI:** 10.1186/1471-2164-11-618

**Published:** 2010-11-05

**Authors:** Víctor M González, Luis Rodríguez-Moreno, Emilio Centeno, Andrej Benjak, Jordi Garcia-Mas, Pere Puigdomènech, Miguel A Aranda

**Affiliations:** 1 Molecular Genetics Department, Center for Research in Agricultural Genomics CRAG (CSIC-IRTA-UAB), Jordi Girona, 18-26, 08034 Barcelona, Spain; 2 Departamento de Biología del Estrés y Patología Vegetal, Centro de Edafología y Biología Aplicada del Segura (CEBAS) - CSIC, Apdo. correos 164, 30100 Espinardo (Murcia), Spain; 3 IRTA, Center for Research in Agricultural Genomics CRAG (CSIC-IRTA-UAB), Carretera de Cabrils Km 2, 08348 (Barcelona), Spain

## Abstract

**Background:**

Although melon (*Cucumis melo *L.) is an economically important fruit crop, no genome-wide sequence information is openly available at the current time. We therefore sequenced BAC-ends representing a total of 33,024 clones, half of them from a previously described melon BAC library generated with restriction endonucleases and the remainder from a new random-shear BAC library.

**Results:**

We generated a total of 47,140 high-quality BAC-end sequences (BES), 91.7% of which were paired-BES. Both libraries were assembled independently and then cross-assembled to obtain a final set of 33,372 non-redundant, high-quality sequences. These were grouped into 6,411 contigs (4.5 Mb) and 26,961 non-assembled BES (14.4 Mb), representing ~4.2% of the melon genome. The sequences were used to screen genomic databases, identifying 7,198 simple sequence repeats (corresponding to one microsatellite every 2.6 kb) and 2,484 additional repeats of which 95.9% represented transposable elements. The sequences were also used to screen expressed sequence tag (EST) databases, revealing 11,372 BES that were homologous to ESTs. This suggests that ~30% of the melon genome consists of coding DNA. We observed regions of microsynteny between melon paired-BES and six other dicotyledonous plant genomes.

**Conclusion:**

The analysis of nearly 50,000 BES from two complementary genomic libraries covered ~4.2% of the melon genome, providing insight into properties such as microsatellite and transposable element distribution, and the percentage of coding DNA. The observed synteny between melon paired-BES and six other plant genomes showed that useful comparative genomic data can be derived through large scale BAC-end sequencing by anchoring a small proportion of the melon genome to other sequenced genomes.

## Background

Melon (*Cucumis melo *L.) is an important horticultural crop grown in temperate, subtropical and tropical regions worldwide. More than 25 million tonnes of fruit were produced in 2007, 64.5% in Asia, 14.6% in Europe, 13.1% in America and 7.8% in Africa [1]. Melon belongs to the *Cucurbitaceae *family, which comprises 90 genera and ~750 species, including other fruit crops such as watermelon (*Citrullus lanatus *(Thunb.) Matsum & Nakai), cucumber (*Cucumis sativus *L.), squash and pumpkin (*Cucurbita *spp.). Genetically, melon is a diploid species (2 × = 2n = 24) with an estimated genome size of 454 Mb [2]. Transgenic melons, first produced in 1990, can now be generated in a range of recalcitrant cultivars [3,4]. Melon fruits are morphologically and biochemically diverse, which makes them particularly suitable for research into the flavor and texture changes that occur during ripening [5].

Despite its economic importance, there are few genomic resources for melon. As of January 2010, 126,940 high-quality expressed sequence tags (ESTs) and 23,762 unigenes were available in public databases [6,7], which is low when compared to the 298,123 ESTs available for tomato (*Solanum lycopersicum *L.) and the 1,249,110 ESTs available for rice (*Oryza sativa *L.) [8]. More recent efforts to increase the availability of genetic and genomic resources for melon [9] have included the construction of bacterial artificial chromosome (BAC) libraries [10,11], the development of oligo-based microarrays [12,13], the production of TILLING and EcoTILLING platforms [14,15] and the development of a collection of near isogenic lines (NILs) [16]. However, the integration of genetic and physical maps is a necessary first step towards sequencing the melon genome, identifying relevant genes using these to discover how economically important aspects of fruit development are controlled [17,18].

Over the last 15 years, several melon genetic maps have been constructed primarily using randomly amplified polymorphic DNAs (RAPDs), restriction fragment length polymorphisms (RFLPs), amplified fragment length polymorphisms (AFLPs) and simple sequence repeats (SSRs) [19-24]. These maps have helped to pinpoint the loci of some important agronomic traits [25-27], but they are sparsely populated and the different markers make them difficult to compare. To address this issue, a genetic map has recently been constructed by merging several of those previous genetic maps [6]. In addition, a melon physical map representing 0.9 × melon genomic equivalents has recently been constructed using both a BAC library and a genetic map previously developed in our laboratory [28]. The physical and genetic maps have been integrated by anchoring 175 genetic markers to the physical map, allowing contigs representing 12% of the melon genome to be anchored to known genetic loci.

It is important to obtain an accurate, representative sample of the genome ahead of full genome sequencing and annotation, and the end-sequencing of large numbers of BAC clones is an efficient strategy to achieve this goal. BAC-end sequences (BES) generate accurate but inexpensive genome samples that give a first impression of properties such as GC content, the distribution of microsatellites and transposable elements, and the amount of coding DNA [29-31]. However, most BAC libraries are constructed by digesting DNA with one or more restriction endonucleases, which introduces a partial bias in coverage because the target sites are distributed in a non-random manner [32]. We therefore sequenced BAC-ends representing 33,024 clones, half from a previously described BAC library generated using restriction endonucleases, but the remainder from a freshly-prepared random shear BAC library to eliminate the possibility of bias. We obtained 47,140 high-quality BES, which were analyzed for GC content, microsatellites, repeat elements and coding regions. A total of 43,224 paired BES were mapped independently onto six sequenced genomes from other dicotyledonous plant species to identify regions of microsynteny.

## Results and discussion

### BAC libraries

Two BAC libraries from the double-haploid melon line PIT92 were used for BAC-end sequencing (Table 1). A BamHI BAC library (BCM) had previously been constructed in our laboratory with an average insert size of 139 kb, representing 5.7 genome equivalents of the melon haploid genome (based on an estimated haploid genome size of 454 Mb [2]) [11]. In order to increase the genome coverage and reduce the bias associated with BAC libraries constructed using non-random DNA fragments, a second BAC library (RCM) was prepared using randomly-sheared melon genomic DNA. With 30,720 BAC clones and an average insert size of 120 kb, this library represents 6.4 genomic equivalents. When combined, the two libraries represent ~12 genomic equivalents.

**Table 1:** Genomic *C. melo *BAC libraries

	BCM	RCM
**Vector**	pECBAC1	pSMART^®^
**Digestion method**	BamHI	Random shear
**Average insert size**	139 kb	120 kb
**No. of clones**	23,040	30,720
**Estimated No. of true clones^1^**	18,432	23,655
**Genomic coverage^2^**	x5.7	x6.4

^1^Non-empty BAC clones containing melon genomic DNA

^2^Based on an estimated haploid genome size of 454 Mb [2]

Fingerprinting of clones from the BCM library allowed the construction of a melon physical map [28]. The analysis of the structure of the BAC contigs revealed a high proportion of 'stacked' contigs, that is, contigs containing regions of depth that exceeded by far the estimated coverage of the library used (5.7×); in fact, most of the contigs contained BACs sharing one border, a feature most probably explained by the unequal use of BamHI restriction sites during library construction. These data strongly suggest that the BCM library could be heavily affected by the non-randomness currently associated to all libraries constructed by one-enzyme restriction of genomic DNA. On the contrary, being a random shear library, RCM should represent more randomly the melon genome, although some bias produced by unequal DNA fragmentation cannot be ruled out.

### BAC end sequencing

We sequenced the ends of 16,512 BCM BACs and 16,512 RCM BACs, generating 23,878 and 23,262 high-quality BES, respectively (Table 2). Together, this amounted to 47,140 high-quality BES, of which 91.7% were paired-BES. The average read length was 543 bp, representing 25.6 Mb of genomic DNA in total or 5.7% of the genome (using the 454 Mb estimate [2]). The BES have been deposited in the GenBank databases under the accession numbers HN291986-HN339125.

**Table 2:** Sequence statistics of the *C. melo *BES

	BCM	RCM	Total
	
Total BES			
Total no. of high-quality BES	23,878	23,262	47,140
paired-BES	21,742	21,482	43,224
non-paired BES	2,136	1,780	3,916
Minimum/maximum length (bp)	50-879	50-874	50-879
Average length (bp)	552	534	543
Total length (Mb)	13.2	12.4	25.6
	**BCM**	**RCM**	**BCM-RCM**
	
**Non-redundant sequences^1^**			
**Total no. Of:**	15,408	19,106	33,372
**contigs**	4,661	1,488	6,411
**singletons**	10,747	17,618	26,961
**Total length (Mb)**	8.9	10.5	18.9
**Minimum/maximum length (bp)**			50/3,473
**Average length (bp)**			658

^1^Sequences obtained by assembly of BCM-BES, RCM-BES or contigs and singletons from the BCM and RCM assemblies

As any analysis based on a highly redundant set of BES would produce unaccurate genomic information, the BCM and RCM BES were assembled independently to reduce sequence redundancy. In order to avoid as much as possible the incorrect assembly of highly repetitive genomic regions, the assembly was performed using rather strict constraints (see Methods section). The BCM assembly produced 4,661 contigs with a total length of 3.1 Mb, as well as 10,747 singletons (5.9 Mb), whereas the RCM assembly produced 1,488 contigs (1.2 Mb) as well as 17,618 singletons (9.2 Mb). Although the numbers of high-quality BES, paired-BES and the average read length were similar for both libraries, only 45% of the BCM BES remained as singletons after assembly, compared to 76% of the RCM BES. Furthermore, the average length of contigs comprising two BES was 631 bp for the BCM assembly and 715 bp for the RCM assembly. These data support the conclusion that the RCM BES are more uniformly distributed across the melon genome than those from the BCM library, as anticipated from the construction methods. The non-random distribution of restriction endonuclease target sites and cytosine methylation targets has promoted research into a number of alternative library construction methods, including the random shearing approach used here and the use of methylation-sensitive partial restriction digests [32-34].

A final combined set of non-redundant, high-quality BES (named BCM-RCM) was produced after the cross-assembly of the BCM and RCM contigs and singletons. This contained 33,372 sequences of average length 568 bp and total length 18.9 Mb, grouped in 6,411 contigs (4.5 Mb) and 26,961 non-assembled BES (14.4 Mb). Only 997 (15%) contigs were longer than the maximum BES length (879 bp). The average GC content was 35.2%, similar to previous values from sequenced melon BAC clones [11,35].

Significantly, the total length of the BCM-RCM assembly was just 430 kb shorter, and contained only 1,142 less sequences, than the global length of the BCM and RCM assemblies on the whole (Table 2). Thus, almost no redundancy seems to exist between BES from the BCM library and those from the RCM library, strongly suggesting that incorrect assembly of BES from duplicate or repetitive genomic regions should not represent a significant reason behind the high redundancy detected when assembling BCM and RCM BES; otherwise, similar levels of redundancy would had been detected when cross-assembling the BCM and RCM contigs and singletons, contrary to our results.

### Simple sequence repeats

The BCM-RCM sequences were screened for SSRs, resulting in the identification of 7,198 microsatellites at least 12 nt in length (1-3 nt repeats) or containing at least four tandem repeat units (4-6 nt repeats). SSRs accounted for 130,222 bp (0.7%) of the total BCM-RCM sequence, which is equivalent to one microsatellite every 2.6 kb (Table 3). Mononucleotide tandem repeats were the most abundant, accounting for 39.7% of all microsatellites, followed by trinucleotides (27.4%) and dinucleotides (24.2%). The poly(A)/poly(T) microsatellite was the most common mononucleotide repeat (94.6%) and the poly(AT) microsatellite was the most common dinucleotide repeat (67.8%). These data confirm previous results from the analysis of two melon BAC clones [11,36] although dinucleotide repeats appear to be less abundant in melon than in many other plants, e.g. apple, tomato, potato, clementine, papaya and banana [29,31,37-39].

**Table 3:** Distribution of SSRs in *C. melo *non-redundant sequences

	Type	Number		
		**BCM**	**RCM**	**BCM - RCM**
	
				
**Monomer**				
	A/T	1,192	1,546	2,700
	C/G	47	110	155
**Dimer**				
	AT	482	710	1,184
	AG/CT	190	250	429
	AC/GT	59	69	128
	CG	4	0	4
**Trimer**				
	AAT/ATT	392	674	1050
	AAG/CTT	217	332	546
	ATC/GAT	47	39	85
	AAC/GTT	31	38	68
	AGG/CCT	36	37	72
	Other	69	90	150
**Tetramer**				
	AAAT/ATTT	65	95	159
	AAAG/CTTT	35	42	75
	AATT	20	21	39
	ACAT/ATGT	5	21	26
	AAAC/GTTT	9	9	17
	Other	18	19	37
**Pentamer**				
	AGCCG/CGGCT	22	62	84
	AAAAG/CTTTT	21	26	47
	AAAAT/ATTTT	13	10	22
	AAAAC/GTTTT	6	6	12
	AATTT/AAATT	3	7	9
	Other	22	16	36
**Hexamer**				
	AAAAAG/CTTTTT	9	6	15
	AAAAAT/ATTTTT	3	3	6
	AAAGAG/CTCTTT	2	1	3
	GAATTT/AAATTC	0	2	2
	Other	15	23	38
		Total: **3,034**	Total: **4,264**	Total: **7,198**
		Total length: 54,754 bp	Total length: 77,267 bp	Total length: 130,222 bp
				
**Length distribution**				
	12 - 20 nt	2,404	3,436	5,759
	21 - 50 nt	5,76	748	1,307
	51 - 100 nt	51	65	114
	> 100 nt	3	15	18

A total of 1,439 (20%) microsatellites were found to be > 20 nucleotides in length (class I microsatellites) making them good candidates for hypervariable, polymorphic markers. The longest microsatellites repeat units of 1-4 nucleotides were a 57-bp poly(T) sequence, a 102-bp poly(AT) repeat, a 465-bp poly(AAT) repeat and a 172-bp poly(ACAT) repeat. The most abundant SSRs for each repeat unit category were generally AT-rich, although exceptionally the most abundant pentanucleotide repeat was AGCCG/CGGCT (representing 40% of all pentanucleotide tandem repeats).

The above analysis was also carried out individually on the BCM and RCM assemblies. When values in Table 3 were normalized relative to the total sequence length of each assembly, RCM was found to contain 1.2 times more SSRs than BCM, with some specific cases (namely C/G, ACAT/ATGT, AGCCG/CGGCT and AATTT/AAATT repeats) showing a marked increase in the microsatellite content of RCM relative to BCM (between x2 for AATTT/AAATT and x3.6 for ACAT/ATGT). Significantly, the number of SSRs longer than 100 nt was found to be four times higher in RCM than in BCM while the number of SSRs shorter than 100 nt was only 1.1 times greater in RCM than in BCM. We therefore conclude that the RCM library represents genomic regions with a higher content and different distribution of microsatellites than those covered by the BCM library.

We validated these results by searching for microsatellites in the melon unigene collection (v3) at ICUGI [6] using identical search parameters. A total of 7,038 SSRs was found among 23,762 unigenes spanning 18.5 Mb of the melon transcriptome. These microsatellites represent 125,363 bp, or 0.7% of the total combined unigene sequence, a density of one SSR every 2.6 kb. Although these figures are equivalent to those obtained using the BCM-RCM assembly, the relative abundance of 1-6 nucleotide repeat microsatellites was quite different, with trinucleotide repeats the most abundant class (47%), followed by mononucleotide (24.8%) and dinucleotide (20.7%) repeats. SSR frequency and type are known to differ between coding DNA, intron DNA and intergenic DNA [40], and it has previously been shown that trinucleotide repeats are the most abundant SSRs in plant coding regions [41].

### Repetitive elements

The BCM-RCM assembly was compared with the plant repeat databases at http://plantrepeats.plantbiology.msu.edu/ and also screened using RepeatMasker software to locate transposable elements, rRNAs and telomere- and centromere-related sequences. A total of 2,484 sequences showed homology with plant repeat elements (representing 6% of the assembly) of which 95.9% were transposable elements (TEs) (Table 4). Class I transposons (retrotransposons) were the most abundant, accounting for 85.4% of TEs. Sequences homologous to retrotransposons were classified as LTR-containing Ty1-Copia (61.6%) and Ty3-Gypsy (34.9%), LINES (1.5%) and other unclassified elements (2%). The next most abundant repeats were class II transposons, 51% of which were classified as En-Spm elements. These figures are similar to those obtained from cucumber, where retrotransposons accounted for 81% of all TEs, the Ty1/Ty3 ratio is 1.4, and En-Spm elements accounted for 54% of all classified DNA transposons [42]. Other plant repeat elements included twelve putative rolling-circle transposons, 105 rDNA sequences and three putative telomere-related sequences.

**Table 4:** Plant repeat element content of *C. melo *non-redundant sequences

		BCM - RCM		
**Class**	**Element**	**No**.	**Total**	**%**

**Retroelements**			**2,030**	3.8
**LINES**		30		
	L1	30		
**LTR**		2,000		
	Ty1/Copia	1,250		
	Ty3/Gypsy	709		
	Unclassified	41		
**DNA transposons**			**335**	1.9
	hobo-Activator	35		
	En-Spm	171		
	MuDR	102		
	Harbinger	5		
	Unclassified	22		
**Rolling circles**			**12**	0.02
	Helitron	12		
**Telomere-related** **Sequences**			**3**	
				
**rRNA genes**			**105**	0.3
	Small subunit	36		
	Large subunit	62		
	45S and Internal spacer region	**7**		
				
**Total no. of** **repeat elements**			**2,484**	6.0

The BCM and RCM assemblies were searched independently for plant repeat sequences and the class I/class II ratio was found to be the same from both sources. However, some differences were detected regarding the relative abundance of specific transposon subfamilies. While En/Spm elements were the most abundant class II transposons in the RCM assembly, accounting for 64% of all DNA transposons and followed by MuDR elements (18%), En/Spm transposons accounted for only 37% of TEs in the BCM assembly, with MuDRs representing 43% of DNA transposons. Also, the Ty1/Ty3 ratios were 2 and 1.5 in the BCM and RCM assemblies, respectively. Significantly, whereas the RCM assembly contained 84 putative rDNA sequences, only 29 were found in the BCM assembly. The genes for the 18*S*, 5.8*S *and 25*S *ribosomal RNAs are present in tandem arrays containing up to 20,000 repeats in a chromosome structure known as the nucleolar organizer region [42]. Genomic regions containing highly repetitive sequences arranged in tandem repeats are often underrepresented in genomic libraries constructed using one restriction endonuclease. Therefore, our data suggest that the melon RCM library represents genomic regions not covered or poorly represented by the BCM library.

### Coding regions

BCM-RCM non-redundant and masked sequences were tested against the 23,762 sequences from the melon unigene database v3 [6] using the BLASTN algorithm (Table 5). Because this approach compares short fragments of genomic DNA against ESTs, the use of stringent match conditions would fail to detect most BES containing intron-exon boundaries, whereas less stringent conditions would increase the number of false positives. As a compromise, a double cut-off approach was used with a low-stringency E value of 10^-20 ^and a high-stringency value of 10^-50^. The low-stringency search identified 7,929 matching sequences, with 4,661 showing > 95% identity to melon unigenes. The remaining unmatched sequences were then used to search other cucurbit unigene databases (81,401 unigenes from the cucumber EST collection v2, and 4,719 unigenes from the watermelon EST collection v1) [6]. A total of 3,064 additional sequences showed homology to those cucurbit unigene sets. Finally, sequences that failed to match any cucurbit EST databases were used to search all non-cucurbit plant EST assembly databases at http://www.plantgdb.org and 379 additional hits were found. In all, 11,372 (34.1%) of the analyzed sequences were shown to contain putative regions of coding DNA. The high-stringency search identified 6,630 hits or 19.9% of all tested sequences. These results suggest that between 20% and 35% of the melon genome consists of coding DNA.

**Table 5:** BLASTN analysis against plant EST databases

Masked and non-redundant *C. melo *sequences *vs*.:	E-value 1 × 10^-20^		E-value 1 × 10^-50^	
	**No. of hits**	**%^1^**	**No. of hits**	**%^1^**
**Melon unigenes^2^**	7,929	23.7	5,072	15.2
**Cucumber/watermelon unigenes^3^**	3,064	9.2	1,478	4.4
**Non-cucurbit plant ESTs^4^**	379	1.1	80	0.2

**Total:**	11,372	34.1	6,630	19.9

^1^Relative to the number of non-redundant *C. melo *BCM-RCM sequences

^2^ICUGI melon_unigen_v3 [6]

^3^ICUGI cucumber_unigen_v2 and watermelon_unigen_v1 [6]

^4^All non-cucurbit plant EST assembly databases from http://www.plantgdb.org

Melon unigene hits accounted for 72.1% of cucurbit hits, or 69.7% of all hits, in the low-stringency search, and for and 77.4% of cucurbit hits, or 76.5% of all hits, in the high-stringency search. This suggests that the current melon unigene database at ICUGI lacks 22-30% of all melon transcripts. Because the melon unigene database contains 23,762 unigenes, we can tentatively estimate that the total size of the melon transcriptome is 27,000-34,000 sequences.

### Comparative mapping of melon BES onto other plant genomes

The analysis of two regions of the melon genome 92 kb and 215 kb in length has previously revealed significant degrees of microsynteny between melon and *Arabidopsis thaliana*, poplar (*Populus trichocarpa*) and *Medicago truncatula *[11,35]. Syntenic relationships facilitate the investigation of genome evolution and dynamics, comparative genomics and phylogeny [43,44], as well as allowing agronomically important genes to be identified and cloned [45]. For example, the melon *nsv *locus, which confers resistance to *Melon necrotic spot virus*, was cloned by exploiting synteny [46].

In order to identify regions of synteny between the melon genome and other sequenced plant genomes, all 47,140 high quality BES were masked for repeats and used to search the genome sequences available in the Phytozome v5.0 database http://www.phytozome.net. Six dicotyledonous species were chosen for the analysis: *A. thaliana*, cucumber, soybean (*Glycine max*), *M. truncatula*, poplar and grapevine (*Vitis vinifera*). Only scaffolds > 500 kb in length were used for comparative mapping in cucumber because the contiguity of the cucumber genome sequence is currently low. The cucumber scaffolds represent 149 Mb or 73.4% of the sequence assembly deposited in the Phytozome database and 61% of the cucumber genome [47]. As shown in Table 6, 6-10% of melon BES matched non-curcubit genomes (9-14% were paired BES), and 65% of melon BES matched the cucumber genome (67% were paired BES). *A. thaliana *showed the lowest number of hits whereas grapevine, poplar and soybean showed similar levels. Because only ~70% of the complete cucumber genome was used, we estimate that more than 90% of all melon BES would show significant homology to the whole cucumber genome sequence if available.

**Table 6:** Comparative mapping of *C. melo *BES to other plant genomes

Plant genomes			No. of masked BES with BLAST matches^1^	No. of masked BES pairs withM BLAST matches^2, 3^	On same chromosome, contig or scaffold^3, 4^		Within 50 - 500 kb^3, 4^		In the correct orientation^3, 4^	%^5^	Total %^6^
**Species**	**Pseudo_** **molecules^7^**	**Length** **(Mb)^8^**				**No. of** **mapping** **Loci^9^**		**No. of** **mapping** **loci**			
									
***A. thaliana***	5	135	2,958 (6.3%)	135 (0.4%)	86 (63.7%)	111 (1.3)	15 (17%)	15	11 (73%)	8	0.05
***V. vinifera***	19	487	4,919 (10.4%)	334 (1.5%)	195 (58.4%)	350 (1.8)	86 (44%)	91	56 (65%)	17	0.26
***G. max***	20	975	4,446 (9.4%)	282 (1.3%)	171 (60.6%)	437 (2.6)	73 (43%)	120	57 (78%)	20	0.26
***M. truncatula***	8	241	3,214 (6.8%)	155 (0.7%)	102 (65.8)	210 (2.0)	30 (29%)	36	24 (80%)	15	0.11
***P. trichocarpa***	19	370	4,676 (9.9%)	315 (1.4%)	171 (54.3%)	276 (1.6)	95 (55%)	123	76 (80%)	24	0.35
***C. sativus*^10^**	117	149	30,818 (65.4%)	10,296 (47.6%)	6,475 (62.9%)	31,385 (4.8)	4,945 (76%)	17,456	4,138 (84%)	40	19.14

^1^Percentage relative to the total number of high-quality BES (47,140)

^2^Percentage relative to the total number of pairs of high-quality BES (43,224/2)

^3^Paired-BES mapping on several genome locations are counted once

^4^Percentage relative to the values in the previous column

^5^Percentage of paired BES with BLAST matches mapping on same chromosome/contig/scaffold within 50-500 kb in the correct orientation

^6^Percentage relative to the total number of paired-BES

^7^Number of pseudomolecules/scaffolds used for the analysis. In all cases except *C. sativus*, pseudomolecules correspond to chromosomes

^8^Total length of the genomic sequences used for the analysis

^9^In parenthesis, average number of mapping loci of pairs of BES mapping on same chromosome/contig/scaffold

^10^Scaffolds > 500 kb, representing 73.4% of the *C. sativus *genome assembly deposited n the Phytozome v5 database.

Synteny between the melon genome and other plants was characterized by searching the BCM-RCM sequence for paired BES that 1) mapped to the same chromosome, 2) within a 50-500 kb region and 3) were oriented correctly with respect to each other. According to these criteria, 0.05-0.35% of all paired BES were deemed potentially collinear with the other plant genomes (except cucumber, see below). Again, *A. thaliana *showed the lowest number of hits (11), followed by *M. truncatula *(24), grapevine (56), soybean (57) and poplar (76). The higher degree of synteny between melon and poplar compared to melon and *M. truncatula*, despite the Cucurbitales being considered phylogenetically closer to Fabales than to Malphigiales, is consistent with previous results from the analysis of two independent melon regions [35]. In cucumber, 4,138 paired BES (19% of all available paired BES) fulfilled the three syntenic conditions listed above. However, the available cucumber genomic sequence is not yet assembled into chromosomes, and the scaffolds > 500 kb are represented by only ~150 sequences. Therefore, a significant number of true collinear paired BES could not be detected due to paired BES mapping into different but adjacent cucumber scaffolds, and so the number of paired BES potentially collinear with the cucumber genome is probably much higher than the above value.

When the requirement for correct orientation was omitted to consider collinear regions that may have suffered localized inversions, there was an increase of 20-56% in the number of paired BES hits. Interestingly, when considering the total number of paired BES that map on the same chromosome, the percentage of paired BES mapping within 50-500 kb is > 40% in all plants except *A. thaliana *and *M. truncatula*. For example, 55% of all paired BES mapping on the same poplar chromosome do so within the prescribed distance. As poplar chromosomes are on average 20 Mb in length, these data show that the mapping loci are not randomly distributed in the poplar genome, which supports our conclusion that our hits reflect true syntenic relationships.

**Figure 1:**
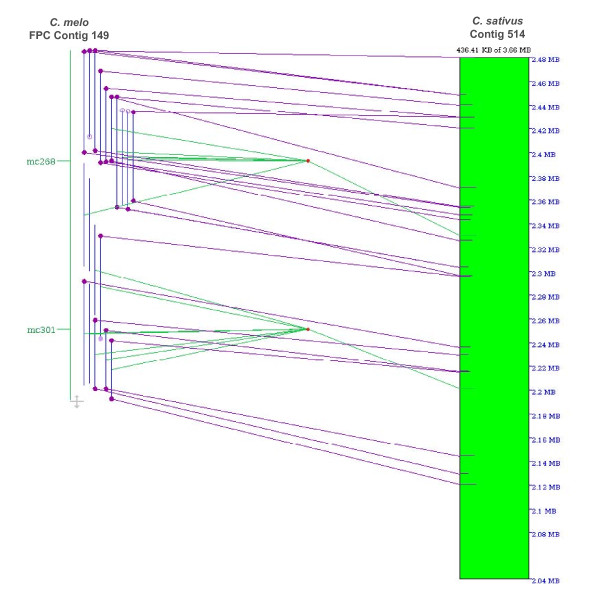
**Microsynteny between contig 149 from the *C. melo *physical map and a 370-kb region of scaffold 542 from the *C. sativus *genome assembly at Phytozome v5.0**. Contig 149 has an estimated size of 514 kb and consists of 19 BACs. There are 28 available BES, represented as full or empty dots at the end of the BACs; full dots represent BES that map to the selected cucumber region whereas empty dots represent BES with no hits. Dark violet dots represent BES with BLAST matches against the cucumber sequence. Violet lines connect BES with the corresponding homologous cucumber regions. Two genetic markers (RFLPs mc268 and mc301) are known to map in the contig 149 region. Green lines show the mapping position of both markers on the cucumber sequence. Additional information regarding the genetic markers, contig 149 and the melon physical map can be found at [29].

Due to the high synteny degree detected between the melon and cucumber genomes, the latter was chosen for an additional synteny analysis performed using the recently published melon physical map of fingerprinted BACs from the BCM library [28]. The cucumber genome assembly together with all BCM BES were tested against the physical map using the draft sequence functions of the FPC software as described in the Methods section. All 320 physical contigs built from at least 15 BAC clones were analyzed and 37.5% were found to contain four of more BES mapping in a contiguous cucumber region greater than 40 kb. A detailed list of the positive melon FCP contigs, cucumber contigs and lengths of the detected synteny regions can be found in the Additional File 1 Table S1. In addition, the SyMAP software package, whose role is to compute and display synteny blocks between physical maps and genome sequences from different species [48,49], was used to obtain graphical representations of the collinear regions between melon and cucumber. Figure 1 shows an example of such a synteny region.

Previous results have shown that the network of microsynteny found between melon and the sequenced genomes of *A. thaliana*, poplar and *M. truncatula *results from the polyploid structure of those genomes, which reflect ancient whole genome duplications [35]. We therefore determined the number of times each BES pair generated hits on the same chromosome (Table 6). In poplar, soybean, grapevine and *M. truncatula*, each BES pair fulfilling the first of the syntenic conditions listed above mapped twice, on average, onto the subject genome. This fell to 1.3 in *A. thaliana *but climbed to 4.8 in cucumber. These figures seem to indicate a genome duplication event in a common ancestor of melon and cucumber as well as another, more ancient event affecting a common ancestor of all the dicotyledonous species we analyzed. We also noted that ~83% of BES pairs mapping to more than one locus mapped to different chromosomes, suggesting a major role of whole-genome/chromosomal duplications rather than intrachromosomal segmental duplications as an explanation for the large number of multiple mapping paired-BES.

The analysis of 348 melon genetic markers suggested that cucumber chromosomes 1, 2, 3, 4, 5 and 6 are collinear with melon chromosomes 2/12, 3/5, 4/6, 9/10, and 8/11, respectively [47]. This suggests that each cucumber chromosome originated from the fusion of two ancestral chromosomes after cucurbit speciation. Several interchromosomal and intrachromosomal rearrangements have also been described [47]. Given the high number of paired melon BES showing collinearity with the cucumber genome, the data presented here will provide further insight into the dynamic evolution of these genomes, particularly once the current low contiguity of the cucumber genome sequence has been addressed.

## Conclusion

We have sequenced BAC-ends from 33,024 clones, half from an existing BAC library produced using restriction endonucleases and the remainder from a newly constructed random shear BAC library. The resulting sequences confirmed that the random shear library is more representative of the melon genome than the restriction library, with less bias against repetitive DNA and fewer gaps. The sequences covered ~4.2% of the melon genome, providing data on the abundance and distribution of microsatellites, TEs and coding DNA, and synteny between melon paired-BES and six other dicotyledonous plant genomes. In particular, we observed sequence conservation and synteny between the melon and cucumber genomes. These results showed that useful comparative genomic information can be derived using a large scale BAC-end sequencing approach by anchoring a small proportion of the melon genome to other sequenced genomes.

## Methods

### Melon genotype and BAC library construction

Young leaves from the double-haploid *C. melo *subsp. *melo *line PIT92 were used for BAC library construction. The BamHI BAC library (BCM) was previously constructed in our laboratory [11]. It comprises 23,040 clones (average insert size 139 kb, 20% empty clones) distributed in 60 384-well plates, representing 5.7 genomic equivalents of the haploid melon genome.

A second BAC library (RCM) was constructed by Lucigen^® ^Co. (Middleton, Wisconsin) using randomly sheared melon genomic DNA. The resulting fragments were size selected and inserted into the transcription-free BAC vector (Lucigen^®^). It comprises 30,720 clones (average insert size 120 kb, 77% clones containing melon nuclear genomic DNA) distributed in 80 384-well plates, representing 6.4 genomic equivalents of the haploid melon genome.

### BAC-end sequencing

BAC-end sequences representing 16,512 clones from the BCM library and 16,512 clones from the RCM library were generated by GATC Biotech (Constance, Germany). The software Phred was used for base calling and sequence trimming. Vector masking was achieved using the Sequencher 4.1.1 software package. BAC clones carrying *Escherichia coli *genomic inserts or melon organellar genomic DNA were removed using BLASTN similarity searches. BES shorter than 50 bp were discarded.

### BAC-end clustering and assembly

Cleaned, high-quality BES from the BCM and RCM libraries were clustered independently using the Sequencher 4.1.1 software package with a minimum overlap of 80, and a minimum match of 95%. The resulting assemblies from both sets of BES were jointly clustered to produce a final set of high-quality and non-redundant sequences. This yielded three sets of non-redundant sequences (referred to as 'BCM', 'RCM' and 'BCM-RCM' sequences) for subsequent analysis.

### Analysis of repetitive elements

Non-redundant sequences were scanned for SSRs using msatcommander 0.8.2. SSRs considered for the final dataset included 1-3 nt repeats at least 12 nt in length, and 4-6 nt repeats with at least four unit repetitions. TEs, telomere-related sequences and rRNAs were identified using RepeatMasker 3.2.8 (parameters: cross-match search engine, default speed/sensitivity and *A. thaliana *DNA source) and the plant repeat databases at http://plantrepeats.plantbiology.msu.edu/ with a cut-off value of 1 × 10^-20^. All identified repeat elements were masked to produce high-quality, non-redundant and masked melon genomic sequences.

### Identification of coding regions

Non-redundant and masked BCM-RCM sequences were tested against the 23,762 sequences in the melon unigene database v3 [6] using E values of 1 × 10^-20 ^and 1 × 10^-50^.

Sequences that did not match the melon database were tested against other cucurbit unigene databases (81,401 unigenes from the cucumber EST collection v2 and 4,719 unigenes from the watermelon EST collection v1) [6] using the same E values. Sequences with no matches in any curcubit EST databases were finally tested against all non-cucurbit plant EST assembly databases found at http://www.plantgdb.org.

### Comparative genome mapping

All BES were mapped against the following genome sequences from the Phytozome v5.0 database http://www.phytozome.net: *A. thaliana *(five chromosomes, TAIR release 9), *P. trichocarpa *(19 chromosomes, from JGI, v2), *M. truncatula *(8 chromosomes, Mt3.0 from the US/EU *M. truncatula *Genome Sequencing Project), *G. max *(20 chromosomes, from the Soybean Genome Project, v1), *V. vinifera *(19 chromosomes, French-Italian Public Consortium for Grapevine Genome Characterization, September 2007 release) and *C. sativus *(117 scaffolds > 500 kb, Roche 454-XLR assembly). The homology searches were performed using Discontiguous Megablast with an E value of 1 × 10^-20^.

### Integration with the melon physical map and representation of synteny

Cleaned, high-quality BES from the BCM library together with the cucumber genome sequence assembly were added to the current *C. melo *FPC physical map [28] using the draft sequence functions of the FPC V9.3 software package http://www.agcol.arizona.edu/software/fpc/ as follows: BSS function using MegaBLAST with E-value 1-20, Identity > 80%, Match > 80%, followed by the Draft Sequence Integration function with parameters: Window size, 200 kb, Min Bes Hit, 4, and Top N, 0. Also, the complete set of BCM BES, the physical map information and the sequences of genetic markers anchored to the map were loaded into the SyMAP v3.1 software package http://www.agcol.arizona.edu/software/symap/ to display areas of synteny between the melon physical map and the sequenced chromosomes of C. sativus.

## Authors' contributions

VMG and LRM carried out the trimming, clustering and analysis of the BAC-end sequences of both BAC libraries and elaborated this manuscript. EC and AB provided bioinformatic analysis support; EC also contributed to the synteny analysis. MAA generated and sent to LUCIGEN^® ^plant melon material for the construction of the second BAC library. PP is the main coordinator of the MELONOMICS project and participated in the conception of the study together with JGM and MAA. MAA is the principal investigator and supervised the writing of the manuscript. All authors read and approved the final manuscript.

## Supplementary Material

Additional file 1 **Table S1**. Mapping of *C. melo *BES to the *C. sativus *genome using the melon FPC physical map. Click here for file
