# Supplementary material for: Genome-wide BAC-end sequencing of Cucumis melo using two BAC libraries
Source: BMC Genomics. 2010 Nov 5;11:618. doi: 10.1186/1471-2164-11-618 (PMC3091759; doi:10.1186/1471-2164-11-618)
Supplement: Additional file 1 — Table S1. Mapping of C. melo BES to the C. sativus genome using the melon FPC physical map. [file 1471-2164-11-618-S1.DOC]

**Table S1: Mapping of *C. melo* BES to the *C. sativus* genome using the melon FPC physical map.^1^**

| ***C. melo***  **FPC contig** | | |  | **C. sativus** | |  | **Mapping region** | |
| --- | --- | --- | --- | --- | --- | --- | --- | --- |
| **Contig** | **Clone Number** | **Estimated length**  **(kb)** |  | **Contig** | **Length**  **(kb)** |  | **BES Number** | **Length**  **(kb)** |
|  |  |  |  |  |  |  |  |  |
| 200 | 143 | 3202 |  | 2532 | 184 |  | 6 | 75 |
| 162 | 86 | 1685 |  | 3703 | 376 |  | 8 | 19 |
| 307 | 69 | 1215 |  | 4158 | 993 |  | 8 | 52 |
|  |  |  |  | 2046 | 871 |  | 10 | 75 |
| 187 | 63 | 1412 |  | 429 | 2602 |  | 11 | 261 |
|  |  |  |  | 227 | 355 |  | 7 | 52 |
| 258 | 49 | 1091 |  | 888 | 995 |  | 11 | 202 |
| 306 | 47 | 1114 |  | 2995 | 3168 |  | 4 | 104 |
| 184 | 46 | 873 |  | 378 | 631 |  | 10 | 188 |
|  |  |  |  | 927 | 3283 |  | 7 | 179 |
| 251 | 46 | 696 |  | 1110 | 1281 |  | 11 | 129 |
| 146 | 45 | 885 |  | 2766 | 516 |  | 8 | 69 |
| 215 | 45 | 589 |  | 2330 | 281 |  | 6 | 95 |
|  |  |  |  | 3537 | 411 |  | 4 | 51 |
| 182 | 43 | 459 |  | 3356 | 5534 |  | 5 | 171 |
| 337 | 40 | 801 |  | 1110 | 1281 |  | 4 | 61 |
| 197 | 39 | 461 |  | 696 | 1757 |  | 10 | 197 |
| 465 | 39 | 691 |  | 3264 | 621 |  | 4 | 41 |
| 185 | 38 | 638 |  | 2229 | 6896 |  | 10 | 182 |
| 243 | 38 | 716 |  | 3611 | 3882 |  | 4 | 50 |
| 280 | 38 | 652 |  | 1037 | 1372 |  | 16 | 387 |
| 98 | 37 | 673 |  | 621 | 669 |  | 5 | 125 |
| 133 | 36 | 550 |  | 540 | 1168 |  | 13 | 150 |
| 368 | 36 | 801 |  | 1236 | 342 |  | 4 | 35 |
|  |  |  |  | 3078 | 624 |  | 6 | 100 |
| 232 | 35 | 776 |  | 2046 | 871 |  | 8 | 223 |
| 2 | 34 | 599 |  | 28 | 4544 |  | 15 | 225 |
| 274 | 34 | 662 |  | 3577 | 2002 |  | 6 | 92 |
| 275 | 34 | 576 |  | 581 | 906 |  | 4 | 147 |
| 363 | 34 | 571 |  | 998 | 3210 |  | 5 | 184 |
| 418 | 34 | 790 |  | 2014 | 156 |  | 11 | 88 |
| 483 | 34 | 674 |  | 793 | 2882 |  | 8 | 82 |
| 1350 | 33 | 599 |  | 3611 | 3882 |  | 6 | 47 |
| 204 | 32 | 643 |  | 154 | 1705 |  | 10 | 124 |
| 387 | 32 | 851 |  | 1227 | 1430 |  | 7 | 210 |
| 131 | 31 | 625 |  | 3625 | 727 |  | 4 | 174 |
|  |  |  |  | 696 | 1757 |  | 4 | 7 |
| 172 | 30 | 571 |  | 2581 | 778 |  | 5 | 108 |
| 546 | 30 | 650 |  | 1079 | 1866 |  | 6 | 87 |
| 36 | 29 | 634 |  | 905 | 410 |  | 6 | 166 |
| 42 | 28 | 604 |  | 3611 | 2882 |  | 4 | 169 |
| 190 | 28 | 324 |  | 998 | 3210 |  | 12 | 53 |
| 302 | 28 | 531 |  | 444 | 902 |  | 4 | 150 |
| 377 | 28 | 637 |  | 1337 | 870 |  | 4 | 93 |
| 1218 | 28 | 500 |  | 1080 | 907 |  | 4 | 87 |
| 526 | 27 | 503 |  | 1154 | 991 |  | 4 | 52 |
| 574 | 27 | 877 |  | 2207 | 528 |  | 7 | 91 |
| 177 | 26 | 391 |  | 1227 | 1430 |  | 8 | 94 |
| 186 | 26 | 529 |  | 581 | 906 |  | 5 | 248 |
|  |  |  |  | 4100 | 1454 |  | 10 | 297 |
| 189 | 26 | 534 |  | 1043 | 432 |  | 5 | 109 |
|  |  |  |  | 789 | 848 |  | 10 | 387 |
| 255 | 26 | 335 |  | 1357 | 1924 |  | 5 | 143 |
| 300 | 26 | 612 |  | 2046 | 871 |  | 8 | 153 |
| 352 | 26 | 519 |  | 3588 | 1653 |  | 19 | 394 |
| 449 | 25 | 716 |  | 3159 | 641 |  | 6 | 50 |
| 555 | 25 | 693 |  | 667 | 108 |  | 5 | 43 |
| 673 | 25 | 553 |  | 540 | 1168 |  | 4 | 91 |
| 163 | 24 | 209 |  | 765 | 949 |  | 8 | 51 |
| 252 | 24 | 409 |  | 429 | 2602 |  | 4 | 122 |
| 263 | 24 | 494 |  | 3577 | 2002 |  | 4 | 80 |
| 271 | 24 | 372 |  | 598 | 204 |  | 9 | 69 |
| 348 | 24 | 372 |  | 2047 | 2614 |  | 7 | 126 |
| 524 | 24 | 614 |  | 927 | 3283 |  | 7 | 176 |
| 147 | 23 | 515 |  | 1225 | 1648 |  | 11 | 223 |
| 266 | 23 | 468 |  | 3578 | 973 |  | 6 | 56 |
| 509 | 23 | 650 |  | 1357 | 1924 |  | 4 | 171 |
| 6 | 22 | 316 |  | 2023 | 1110 |  | 9 | 174 |
| 25 | 22 | 491 |  | 1079 | 1866 |  | 9 | 136 |
| 70 | 22 | 387 |  | 3611 | 3882 |  | 8 | 100 |
| 378 | 22 | 479 |  | 926 | 982 |  | 13 | 132 |
| 460 | 22 | 678 |  | 953 | 1831 |  | 13 | 195 |
| 44 | 21 | 388 |  | 919 | 2735 |  | 16 | 288 |
| 210 | 21 | 398 |  | 999 | 676 |  | 7 | 184 |
| 234 | 21 | 413 |  | 429 | 2602 |  | 11 | 264 |
| 74 | 20 | 583 |  | 1071 | 701 |  | 12 | 157 |
| 217 | 20 | 247 |  | 2511 | 915 |  | 6 | 54 |
| 445 | 20 | 414 |  | 614 | 1267 |  | 6 | 44 |
| 626 | 20 | 438 |  | 154 | 1705 |  | 8 | 44 |
| 1340 | 20 | 324 |  | 1443 | 579 |  | 5 | 57 |
| 1342 | 20 | 344 |  | 1153 | 1691 |  | 18 | 176 |
| 143 | 19 | 509 |  | 1044 | 1435 |  | 4 | 127 |
| 149 | 19 | 514 |  | 542 | 3656 |  | 14 | 319 |
| 225 | 19 | 378 |  | 1236 | 342 |  | 7 | 98 |
| 313 | 19 | 631 |  | 3577 | 2002 |  | 7 | 113 |
| 318 | 19 | 304 |  | 2229 | 6896 |  | 12 | 194 |
| 1352 | 19 | 278 |  | 3356 | 5534 |  | 5 | 98 |
| 9 | 18 | 382 |  | 3443 | 336 |  | 8 | 140 |
| 67 | 18 | 491 |  | 1376 | 498 |  | 12 | 267 |
| 164 | 18 | 268 |  | 862 | 661 |  | 7 | 77 |
| 399 | 18 | 397 |  | 1029 | 887 |  | 5 | 78 |
| 408 | 18 | 649 |  | 3733 | 299 |  | 4 | 149 |
| 429 | 18 | 221 |  | 2229 | 6896 |  | 12 | 188 |
| 554 | 18 | 386 |  | 1028 | 582 |  | 6 | 211 |
| 607 | 18 | 376 |  | 926 | 982 |  | 6 | 79 |
| 71 | 17 | 476 |  | 3588 | 1653 |  | 11 | 182 |
| 119 | 17 | 330 |  | 1357 | 1924 |  | 4 | 128 |
| 151 | 17 | 472 |  | 154 | 1705 |  | 4 | 46 |
| 183 | 17 | 375 |  | 696 | 1757 |  | 6 | 84 |
| 253 | 17 | 371 |  | 602 | 460 |  | 5 | 74 |
| 317 | 17 | 316 |  | 789 | 848 |  | 8 | 180 |
| 366 | 17 | 325 |  | 1221 | 612 |  | 7 | 55 |
| 374 | 17 | 330 |  | 1139 | 763 |  | 8 | 55 |
| 462 | 17 | 453 |  | 998 | 3210 |  | 4 | 67 |
| 697 | 17 | 432 |  | 791 | 799 |  | 4 | 107 |
| 101 | 16 | 529 |  | 542 | 3656 |  | 4 | 61 |
| 111 | 16 | 337 |  | 48 | 139 |  | 7 | 114 |
| 161 | 16 | 270 |  | 894 | 2799 |  | 11 | 146 |
| 179 | 16 | 285 |  | 953 | 1831 |  | 7 | 190 |
| 191 | 16 | 301 |  | 1119 | 545 |  | 5 | 85 |
| 242 | 16 | 344 |  | 3223 | 176 |  | 4 | 75 |
| 618 | 16 | 426 |  | 540 | 1168 |  | 7 | 76 |
| 645 | 16 | 387 |  | 1001 | 872 |  | 7 | 133 |
| 878 | 16 | 352 |  | 4158 | 993 |  | 5 | 81 |
| 68 | 15 | 279 |  | 2035 | 2614 |  | 4 | 125 |
| 81 | 15 | 440 |  | 542 | 3656 |  | 7 | 337 |
| 124 | 15 | 318 |  | 2653 | 2767 |  | 13 | 211 |
| 238 | 15 | 270 |  | 1004 | 301 |  | 4 | 42 |
| 248 | 15 | 175 |  | 894 | 2799 |  | 11 | 49 |
| 273 | 15 | 316 |  | 2229 | 6896 |  | 11 | 222 |
| 346 | 15 | 256 |  | 1233 | 558 |  | 5 | 105 |
| 450 | 15 | 548 |  | 1001 | 872 |  | 4 | 115 |
| 536 | 15 | 302 |  | 2229 | 6896 |  | 8 | 132 |
| 541 | 15 | 310 |  | 1024 | 584 |  | 5 | 115 |
| 759 | 15 | 344 |  | 3611 | 3882 |  | 11 | 141 |
| 779 | 15 | 471 |  | 3882 | 1029 |  | 6 | 55 |
| 850 | 15 | 482 |  | 3312 | 335 |  | 11 | 126 |
| 1341 | 15 | 324 |  | 1357 | 1924 |  | 8 | 207 |

^1^All 320 *C. melo* FPC contigs built from at least 15 BAC clones were analyzed for the presence of a minimum of 4 BAC-ends mapping in a contiguous region longer than 40 kb of the *C. sativus* genome. The melon physical map information can be found in: González V, Garcia-Mas J, Arús P, Puigdomènech P: **Generation of a BAC-based physical map of the melon genome**. *BMC Genomics* 2010, **11**: 339. The cucumber contig sequence information can be found in [www.phytozome.net](../Additional%20file%201.doc) (Roche 454-XLR cucumber genome assembly).
